# The Impact of Climate Change on the Resistance of Rice Near-Isogenic Lines with Resistance Genes Against Brown Planthopper

**DOI:** 10.1186/s12284-021-00508-6

**Published:** 2021-08-02

**Authors:** Yun-Hung Kuang, Yu-Fu Fang, Shau-Ching Lin, Shin-Fu Tsai, Zhi-Wei Yang, Charng-Pei Li, Shou-Horng Huang, Sherry Lou Hechanova, Kshirod K. Jena, Wen-Po Chuang

**Affiliations:** 1 Department of Agronomy, National Taiwan University, Taipei, 10617 Taiwan; 2 Crop Improvement Division, Taoyuan District Agricultural Research and Extension Station, Council of Agriculture, Taoyuan City, 32745 Taiwan; 3 Crop Science Division, Taiwan Agricultural Research Institute, Council of Agriculture, Taichung City, 41362 Taiwan; 4 Department of Plant Protection, Chiayi Agricultural Experiment Station, Taiwan Agricultural Research Institute, Council of Agriculture, Chiayi, 60044 Taiwan; 5 Novel Gene Resources Laboratory, Strategic Innovation Platform, International Rice Research Institute, DAPO Box 7777, Metro Manila, Los Baños, Philippines; 6 School of Biotechnology, Kalinga Institute of Industrial Technology, Bhubaneswar, Odisha 751024 India

**Keywords:** *Nilaparvata lugens*, Near-isogenic lines, Climate change, Insect resistance gene, Host plant resistance

## Abstract

**Background:**

The impact of climate change on insect resistance genes is elusive. Hence, we investigated the responses of rice near-isogenic lines (NILs) that carry resistance genes against brown planthopper (BPH) under different environmental conditions.

**Results:**

We tested these NILs under three environmental settings (the atmospheric temperature with corresponding carbon dioxide at the ambient, year 2050 and year 2100) based on the Intergovernmental Panel on Climate Change prediction. Comparing between different environments, two of nine NILs that carried a single BPH-resistant gene maintained their resistance under the environmental changes, whereas two of three NILs showed gene pyramiding with two maintained BPH resistance genes despite the environmental changes. In addition, two NILs (NIL-*BPH17* and NIL-*BPH20*) were examined in their antibiosis and antixenosis effects under these environmental changes. BPH showed different responses to these two NILs, where the inhibitory effect of NIL-*BPH17* on the BPH growth and development was unaffected, while NIL-*BPH20* may have lost its resistance during the environmental changes.

**Conclusion:**

Our results indicate that BPH resistance genes could be affected by climate change. NIL-*BPH17* has a strong inhibitory effect on BPH feeding on phloem and would be unaffected by environmental changes, while NIL-*BPH20* would lose its ability during the environmental changes.

## Background

Rice (*Oryza sativa* L.) is an important staple cereal crop in Asian countries. It feeds more than half of the world’s population (Jena and Kim 2020), in which global rice production is approximately 782 million tons (FAOSTAT 2020). However, rice pests, such as *Nilaparvata lugens* Stål, *Nephotettix virescens* Distant, *Sogatella furcifera* Horváth, *Chilo suppressalis* Walker, and *Cnaphalocrocis medinalis* Guenée, have been causing a severe impact on rice production, resulting in a potential 13% to 26% yield loss (Oerke 2006). *N. lugens* (brown planthopper; BPH) is the most destructive rice pest in Asia. *N. lugens* directly damages the crop by sucking the phloem sap and causes a plant mortality symptom called the “hooper burn.” During feeding, *N. lugens* could transmit the grassy and ragged stunt viruses to the rice plant. Millions of dollars have been lost due to the *N. lugens* infestation in rice in Southeast Asia (Herdt 1991).

In addition, more than 1.8 trillion tons of carbon dioxide (CO_2_) have been released into the atmosphere due to human-related activity since the Industrial Revolution (Allen et al. 2009), with the greenhouse gas, CO_2_, increasing the atmospheric temperature of the earth. The Intergovernmental Panel on Climate Change (IPCC) predicted that the atmospheric temperature will increase by at least 1.5 °C before 2030 and 2 °C before 2050 (Stocker et al. 2013). Furthermore, climate change would increase the frequency of extreme weather events, such as droughts, water shortages, floods, and typhoons. In addition to the environmental stresses, crop plants may have a higher frequency of insect herbivory damage. It is also predicted that global warming would increase the insect population size and metabolic rates, which could cause substantial crop yield losses (Deutsch et al. 2018). Thus, climate change is predicted to have a severe impact on the staple food production and food quality.

Planting insect-resistant crops is one of the main strategies for integrated pest management (IPM). In rice, more than 30 BPH-resistant genes have been identified (Du et al. 2020), where the *BPH4* gene was identified in Babawee (Sidhu and Khush 1979), while the dominant gene, *BPH9,* was identified in the rice cultivars Kaharmana, Balamawee, and Pokkali (Murata et al. 2001). The *BPH10* gene was found in an introgression line from *O. autraliensis* (Ishii et al. 1994)*,* while *BPH17* was identified from the Sri Lankan indica rice cultivar, Rathu Heenati (Sun et al. 2005). In addition, *BPH18* was found in *O. australiensis* (Jena et al. 2006)*,* while *BPH20* and *BPH21* were identified from *O. minuta* (Rahman et al. 2009)*.* Moreover, *BPH26* was identified from the indica cultivar ADR52 (Myint et al. 2012), in which *BPH32* (old name *BPH3*) was identified in the rice variety PTB33 (Jairin et al. 2006; Ren et al. 2016). In addition, 14 BPH genes (*BPH1*, *BPH2*, *BPH3*, *BPH6*, *BPH7*, *BPH9*, *BPH10*, *BPH14*, *BPH15*, *BPH18*, *BPH21*, *BPH26*, *BPH29*, and *BPH32*) have been cloned (Du et al. 2009; Jena et al. 2017; Ji et al. 2016; Liu et al. 2015; Ren et al. 2016; Tamura et al. 2014; Wang et al. 2015; Zhao et al. 2016). Currently, many BPH-resistant genes have been used to develop insect-resistant varieties through marker-assisted selection.

Although many BPH resistance genes have been identified, a BPH-resistant rice variety would probably be overcome by *N. lugens* within a few years after being released into the market, since *N. lugens* has multiple biotypes and is prone to gain resistance (Stout and Davis 2009). Based on the studies of the resistance responses of *N. lugens* biotypes, BPH-resistant varieties gradually lose their resistance (Cheng and Chang 1979; Huang et al. 2009; Smith 2005). Furthermore, temperature affects the virus or insect resistance in plants (Fahim et al. 2012; Wang et al. 2010). Several virus-resistant genes in wheat would lose their resistance under high temperatures (18 °C) (Fahim et al. 2012), where two insect resistant varieties (IR26 and IR36) that carried a single BPH-resistant gene lost their resistance when the temperature increased up to 31 °C (Wang et al. 2010). To date, the number of BPH-resistant genes that have been affected by environmental changes is unclear.

Furthermore, reducing pesticide usage without decreasing the sustainable crop production is a major challenge. Unfortunately, global pesticide sales increased dramatically from 2000 to 2012 in Asia (Lamichhane et al. 2016). Thus, planting the insect-resistant variety instead of spreading the pesticide is a better strategy to sustain the planet. However, in order to keep the insect-resistant variety sustainable, the impact of climate change on these insect-resistant varieties needs to be considered. The success of using insect-resistant genes is based on the understanding of whether these genes would maintain their resistance traits under climate change. Otherwise, the misuse of insect-resistant genes will not only have no gain in crop production, but also potentially cause the loss of resistance. Hence, in this study, a series of rice near-isogenic lines (NILs) carrying BPH-resistant genes (*BPH4*, *BPH9*, *BPH10*, *BPH17*, *BPH18*, *BPH20*, *BPH21*, *BPH26*, *BPH32, BPH2 + 32, BPH18 + 32,* and *BPH9 + 32*) (Jena et al. 2017) (Table 1) from the International Rice Research Institute (IRRI) were tested for their resistance responses under three environmental conditions (the atmospheric temperature with corresponding carbon dioxide at the *ambient*, year *2050* and the year *2100*) based on the IPCC prediction (Stocker et al. 2013). The standard seed-box screening test (SSST) as well as examining the antibiotic and antixenosis effects of these NILs was performed to evaluate the resistance under environmental changes. The results of this study would provide information to rice breeders for future breeding programs to implement IPM strategies.

**Table 1:** The chromosome number and linked markers of BPH resistant genes in NILs

NILs	BPH resistance gene	Chromosome	Linked markers	References
NIL-*BPH4*	*BPH4*	6	RM589, RM586, RM190	(Jairin et al. 2010)
NIL-*BPH9*	*BPH9*	12	RM5341,RM463 RM28502, InD2	(Su et al. 2006)
NIL*-BPH10*	*BPH10*	12	RG457,RM277, RM260	(Ishii et al. 1994)
NIL-*BPH17*	*BPH17*	4	RM518,RM8213, RM5953,RM401	(Sun et al. 2005)
NIL-*BPH18*	*BPH18*	12	7312.T4A, BPH18-ind2	(Jena et al. 2006)
NIL-*BPH20*	*BPH20*	4	S4019	(Rahman et al. 2009)
NIL-*BPH21*	*BPH21*	12	S12094	(Rahman et al. 2009)
NIL-*BPH26*	*BPH26*	12	RM309, RM28449, S20103, RM5479, MSSR2	(Yara et al. 2010)
NIL-*BPH32*	*BPH32*	6	RM589, RM588, RM8072, PASH6	(Ren et al. 2016)
NIL-*BPH2 + 32*	*BPH2/BPH32*	12/6	RM463, RM3331, RM589, RM588, RM8072, PASH6	(Murata et al. 1998; Ren et al. 2016)
NIL-*BPH18 + 32*	*BPH18/BPH32*	12/6	7312.T4A, BPH18-ind2, RM463, RM3331, RM589, RM588, RM8072, PASH6	(Jena et al. 2006; Ren et al. 2016)
NIL-*BPH9 + 32*	*BPH9/BPH32*	12/6	RM5341, RM463, RM28502, InD2, RM589, RM588, RM8072, PASH6	(Su et al. 2006; Zhao et al. 2016)
IR24	–			

## Results

### SSST under Climate Change

A total of 12 NILs and IR24 (background cultivar) were evaluated in their ability to resist *N. lugens* under three environmental conditions (ambient, 2050, and 2100) using the SSST (Table 2). A two-way ANOVA was used to analyze the damages across the different environmental settings, where the damage score was found to be significantly affected by variety (*P* < 0.001) and environment (*P* = 0.006) (Table 3). Under the ambient setting, 11 NILs (NIL-*BPH4*, NIL-*BPH9*, NIL-*BPH10*, NIL-*BPH17*, NIL-*BPH18*, NIL-*BPH20*, NIL-*NPH21*, NIL-*BPH26*, NIL-*BPH2 + 32*, NIL-*BPH18 + 32*, and NIL-*BPH9 + 32*) had a lower damage score compared to IR24 (Table 2), while NIL-*BPH32* had a high damage score, which was similar to that of IR24. Under the 2050 setting, five NILs (NIL-*BPH17*, NIL-*BPH20*, NIL-*BPH2 + 32*, NIL-*BPH18 + 32*, and NIL-*BPH9 + 32*) had a lower damage score compared to IR24 (Table 2), while seven NILs (NIL-*BPH4*, NIL-*BPH9*, NIL-*BPH10*, NIL-*BPH18*, NIL-*BPH21*, NIL-*BPH26*, and NIL-*BPH32*) had a high damage score, which was similar to that of IR24. However, under the 2100 setting, six NILs (NIL-*BPH17*, NIL-*BPH20*, NIL-*BPH26*, NIL-*BPH32*, NIL-*BPH18 + 32*, and NIL-*BPH9 + 32*) had a lower damage score compared to IR24 (Table 2), while six NILs (NIL-*BPH4*, NIL-*BPH9*, NIL-*BPH10*, NIL-*BPH18*, NIL-*BPH21*, and NIL-*BPH2 + 32*) had a high damage score, which was similar to that of IR24. Hence, these results indicate that some of the BPH resistance genes may have been affected by the environmental changes.

**Table 2:** The SSST of NILs under three different environments

Varieties /NILs	TN1	IR24	NIL- ***BPH4***	NIL- ***BPH9***	NIL-***BPH10***	NIL-***BPH17***	NIL-***BPH18***	NIL-***BPH20***	NIL-***BPH21***	NIL-***BPH26***	NIL-***BPH32***	NIL-***BPH2 + 32***	NIL-***BPH18 + 32***	NIL-***BPH9 + 32***
Environment														
***Ambient***	9.00 ± 0.00 a	8.20 ± 0.20 ab	6.00 ± 0.91 defg	4.75 ± 0.75 ghi	6.75 ± 0.75 cdef	2.40 ± 0.60 klm	5.67 ± 0.33 efgh	4.00 ± 0.58 hijk	5.33 ± 0.88 fghi	5.67 ± 1.33 efgh	7.33 ± 0.33 bcde	5.67 ± 0.88 efgh	2.67 ± 1.20 klm	2.33 ± 0.88 klm
***2050***	9.00 ± 0.00 a	7.78 ± 0.28 bc	6.33 ± 0.88 cdefg	7.00 ± 0.58 bcdef	8.00 ± 0.58 abc	2.33 ± 0.67 klm	7.33 ± 0.33 bcde	5.67 ± 0.33 efgh	7.33 ± 0.67 bcde	6.67 ± 0.67 cdef	6.33 ± 0.67 cdefg	5.67 ± 0.33 efgh	2.33 ± 0.33 klm	2.00 ± 0.58 lm
***2100***	9.00 ± 0.00 a	7.44 ± 0.41 bcd	8.00 ± 0.58 abc	7.67 ± 0.67 bcd	8.67 ± 0.33 ab	1.33 ± 0.67 m	8.00 ± 0.58 abc	5.67 ± 1.33 efgh	7.67 ± 0.88 bcd	5.33 ± 0.33 fghi	4.67 ± 1.33 ghij	6.00 ± 0.58 defg	3.67 ± 1.33 ijkl	3.00 ± 1.15 jklm

The damage score of *N. lugens* nymphs fed on the TN1, IR24, and NILs based on the standard evaluation method (IRRI 2013). Means followed by different letters differ significantly (*P* < 0.05)

**Table 3:** Two-way ANOVA on the SSST result (damage score) of NILs responses to multiple factors

Source of variation	Df	SS	F-value	*P*-value
Environment^a^	2	2.7294	1.0190	0.3639
Variety^b^	13	251.9259	14.4706	< 0.0001***
Environment X Variety	26	70.0278	2.0112	0.0058**
Residuals	127	170.0778		

^a^*Ambient*, *2050*, *2100*

^b^TN1, IR24, NIL-*BPH4,* NIL-*BPH9,* NIL-*BPH10,* NIL-*BPH17,* NIL-*BPH18,* NIL-*BPH20,* NIL-*BPH21,* NIL-*BPH26,* NIL-*BPH32,* NIL-*BPH2 + 32,* NIL-*BPH18 + 32,* NIL-*BPH9 + 32*

**P < 0.01, ***P < 0.001

When comparing across the different environments, eight NILs had changed their resistance level under the environmental changes. Within these NILs, five of them (NIL-*BPH4*, NIL-*BPH9*, NIL-*BPH10*, NIL-*bph18*, and NIL-*BPH21*) were resistant in the ambient setting, but they lost their resistance under the 2050 and 2100 settings. NIL-*BPH26* was resistant in the ambient and 2100 settings, but lost its resistance by 2050. In addition, the NIL-*BPH2 + 32* showed resistance under the ambient and 2050 settings, but lost its resistance under the 2100 setting. Furthermore, NIL-*BPH32* showed no resistance under the ambient and 2050 settings, but it regained its resistance under the 2100 setting. Moreover, four NILs (NIL-*BPH17*, NIL-*BPH20*, NIL-*BPH18 + 32*, and NIL-*BPH9 + 32*) were unaffected by *N. lugens*, and hence, were resistant to the environmental changes. Overall, two of the nine NILs that carry a single BPH resistance gene maintained their resistance to the environmental changes, whereas two of the three NIL gene pyramids with two BPH resistance genes maintained their resistance under the environmental changes.

### The Antibiosis and Antixenosis effects of NIL-BPH17 & NIL-BPH20 on *N. lugens*.

**Fig. 1:**
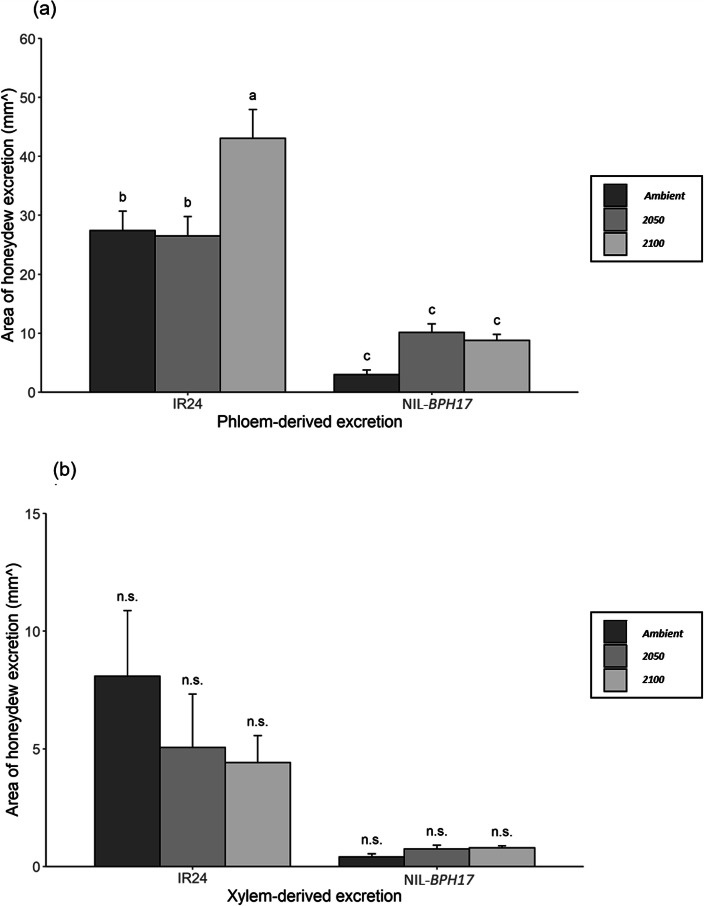
Areas of honeydew excretion of *N. lugens* female feeding on IR24 and NIL-*BPH17* under different environments. **a** Phloem-derived excretion. **b**Xylem-derived excretion. Means in each column followed by a different letter differ significantly (*P* < 0.05)

**Fig. 2:**
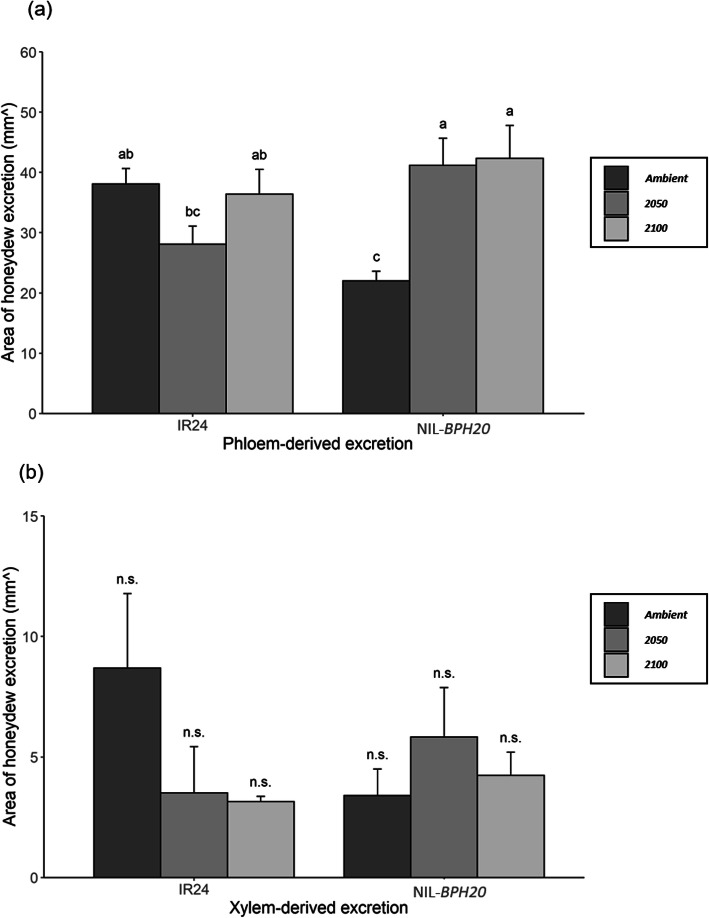
Areas of honeydew excretion of *N. lugens* female feeding on IR24 and NIL-*BPH20* under different environments. **a** Phloem-derived excretion. **b**Xylem-derived excretion. Means in each column followed by a different letter differ significantly (*P* < 0.05)

Based on the results of SSST (Table 2), two NILs that carry a single BPH-resistant gene (NIL-*BPH17* and NIL-*BPH20*) were chosen to test for the antibiosis and antixenosis effects. The honeydew excretion, PGR, nymph survival rate, and oviposition bioassay were used to evaluate the antibiosis effect, while the choice test was used to evaluate the antixenosis effect. Honeydew production was measured using a filter paper with bromocresol green, where the areas of phloem and xylem-derived excretions were further calculated. Compared to IR24 and NIL-*BPH17*, *N. lugens* showed a lower phloem sap consumption in NIL-*BPH17* than in IR24 (Fig. 1a), whereas there was no difference in the xylem sap consumption (Fig. 1b). In the case of NIL-*BPH17*, there was no environmental effect that was observed on the phloem and xylem sap consumption (Fig. 1). When comparing with IR24 and NIL-*BPH20*, *N. lugens* had a lower phloem sap consumption in NIL-*BPH20* than in IR24 under the ambient setting (Fig. 2a), while *N. lugens* had a higher phloem sap consumption in NIL-*BPH20* than in IR24 under the 2050 setting (Fig. 2a) and no difference was noted under the 2100 setting (Fig. 2a). In addition, there was no difference in the xylem sap consumption (Fig. 2b). In the case of IR24, there was no environmental effect that was observed on the phloem and xylem sap consumption (Fig. 2). In NIL-*BPH20*, *N. lugens* had a higher phloem sap consumption under the 2050 and 2100 settings than in the ambient (Fig. 2a). However, there was no environmental effect on the xylem sap consumption in NIL-*BPH20* (Fig. 2b). Hence, these results revealed that NIL-*BPH17* has a strong inhibitory effect on the *N. lugens* feeding on phloem and would be unaffected by environmental changes, while NIL-*BPH20* would lose its ability to inhibit the environmental changes.

**Fig. 3:**
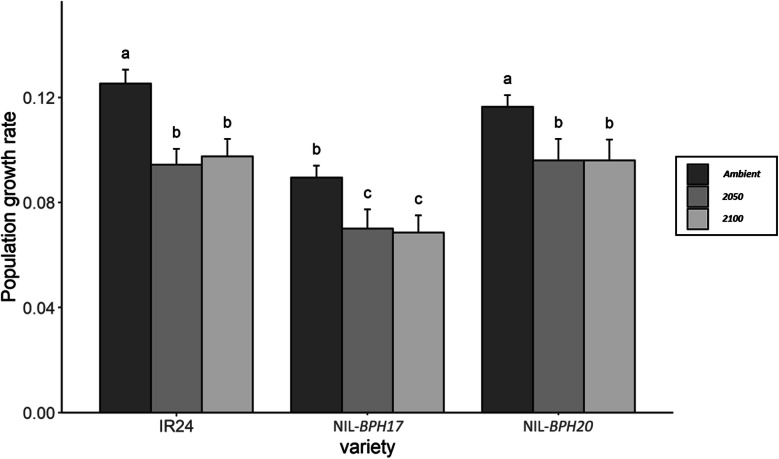
The population growth rate of *N. lugens* nymphs feeding on IR24 and NILs under different environments. **a** IR24 and NIL-*BPH17*. **b** IR24 and NIL-*BPH20*. Means in each column followed by a different letter differ significantly (*P* < 0.05)

**Fig. 4:**
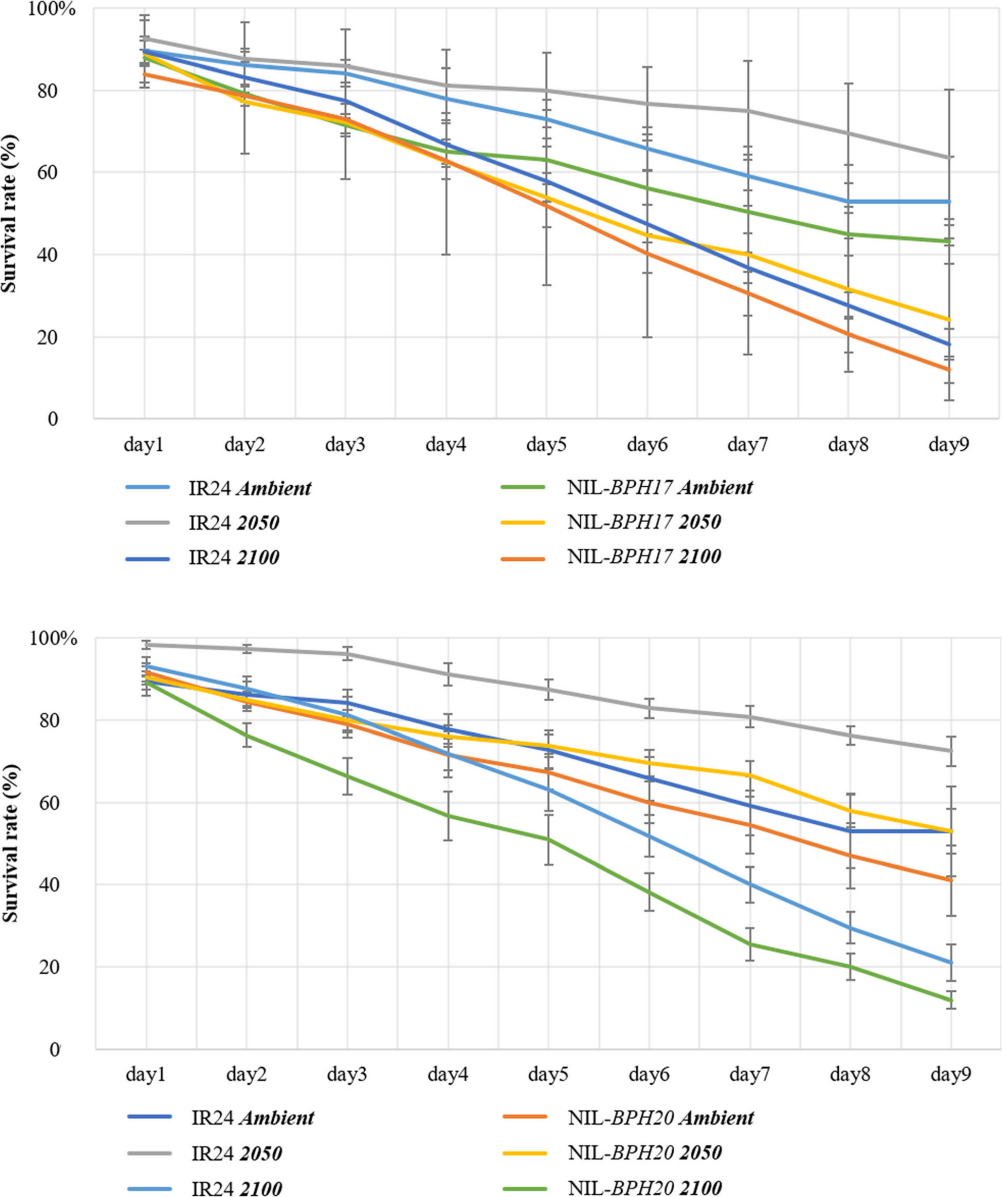
The nymph survival rate of *N. lugens* feeding on IR24 and NILs under different environments. **a** IR24 and NIL-*BPH17*. **b** IR24 and NIL-*BPH20*

Since PGR was one of the parameters used to determine the *N. lugens* growth and development (Du et al. 2009; Qiu et al. 2012), when compared with IR24, NIL-*BPH17* and NIL-*BPH20*, *N. lugens* has a lower PGR in NIL-*BPH17* than in IR24 and NIL-*BPH20* under each environment (Fig. 3). In addition, there was no difference between IR24 and NIL-*BPH20* in terms of the *N. lugens* PGR under each environmental setting (Fig. 3). When comparing across the environmental conditions, the *N. lugens* PGR was lower in 2050 and 2100 than in the ambient setting (Fig. 3). These results indicate that the environment in the future would decrease *N. lugens* growth and development. To further understand the antibiosis effect on NILs, the nymph survival rate of *N. lugens* was also measured (Fig. 4). Based on the 9-day results, the *N. lugens* nymph survival rate was affected by the environment, variety, and the interaction between environment and variety (Table 4).

**Table 4:** Two-way ANOVA on the day 9 survival rate of *N. lugens* nymphs feeding on IR24, NIL-*BPH17* and NIL-*BPH20* responses to multiple factors

Source of variation	Df	SS	F-value	*P*-value
Environment^a^	2	0.6664	9.3726	0.0002***
Variety^b^	2	1.0233	14.3924	< 0.0001***
Environment X Variety	4	0.5429	3.8176	0.0063**
Residuals	97	3.4483		

^a^*Ambient*, *2050*, *2100*

^b^IR24, NIL-*BPH17,* NIL-*BPH20*

**P < 0.01, ***P < 0.001

**Fig. 5:**
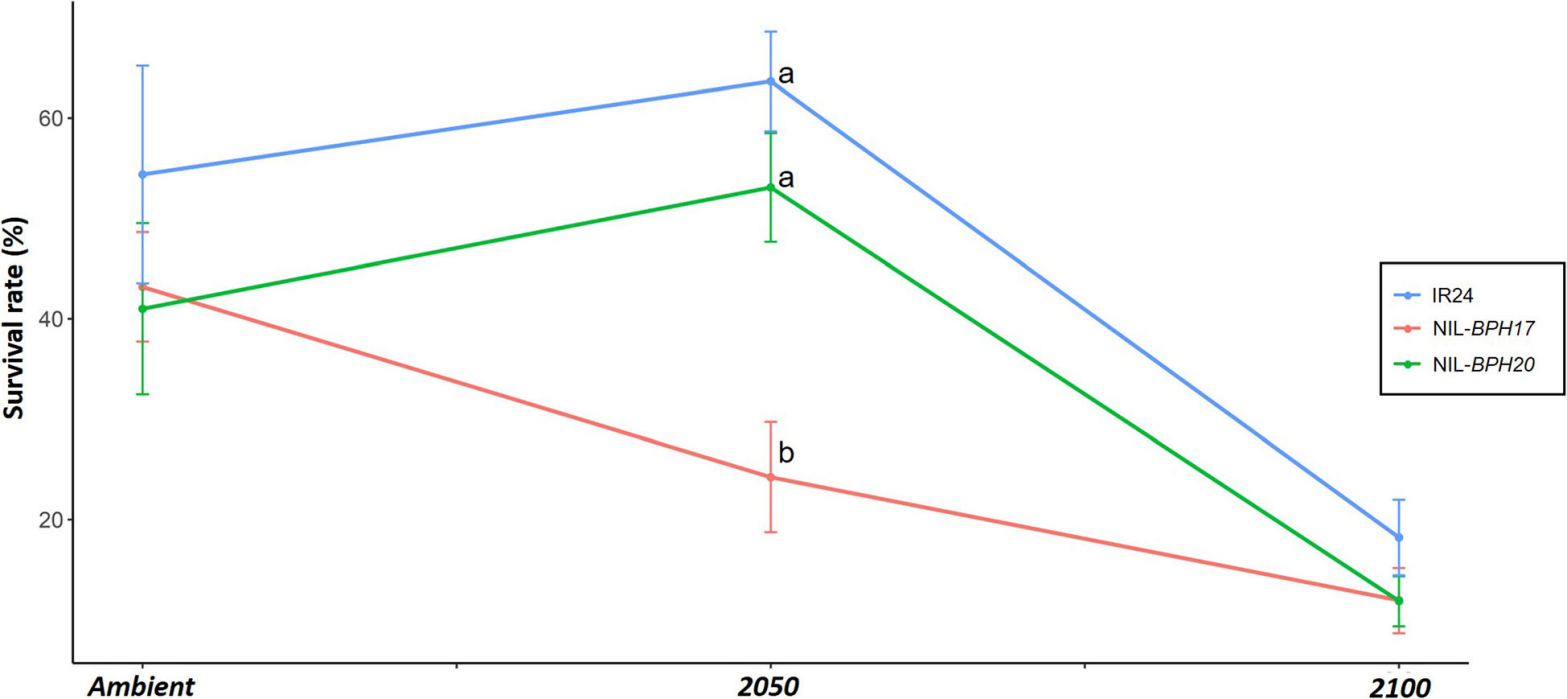
The interaction plot of day 9 survival rate of *N. lugens* nymphs under different environments. Means in each column followed by a different letter differ significantly (*P* < 0.05)

Within the NILs, the nymph survival rate of *N. lugens* feeding on NIL-*BPH17* was lower than that of *N. lugens* feeding on IR24 and NIL-*BPH20* (*P* < 0.001) (Table 4), while in terms of the environments, the *N. lugens* nymph survival rate was lower at 2100 than in the ambient and 2050 settings (*P* < 0.001) (Table 4), which indicates that the environment of 2100 may not be suitable for *N. lugens* nymphs. Furthermore, the nymph survival rate of *N. lugens* on NIL-*BPH17* was different from that of IR24 and NIL-*BPH20* under the environmental changes (Fig. 5). When the atmospheric temperature and carbon dioxide concentrations were increased, NIL-*BPH17* had a stronger resistance against the *N. lugens* nymphs (Fig. 5), in which NIL-*BPH20* showed the same trend as IR24 (Fig. 5).

**Fig. 6:**
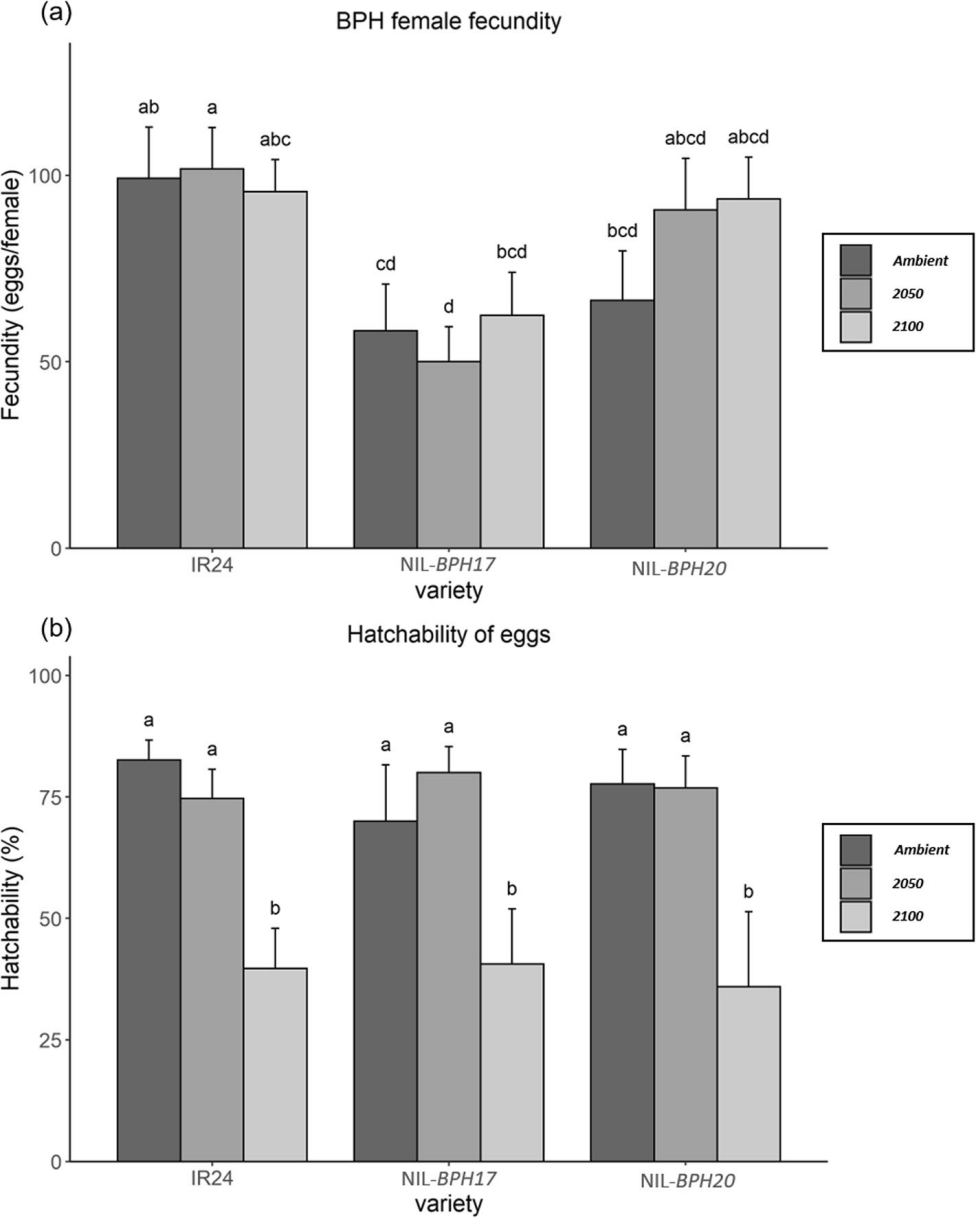
The *N. lugens* female fecundity and hatchability of IR24 and NILs under different environments. **a** Fecundity. **b** Hatchability. Means in each column followed by a different letter differ significantly (*P* < 0.05)

**Fig. 7:**
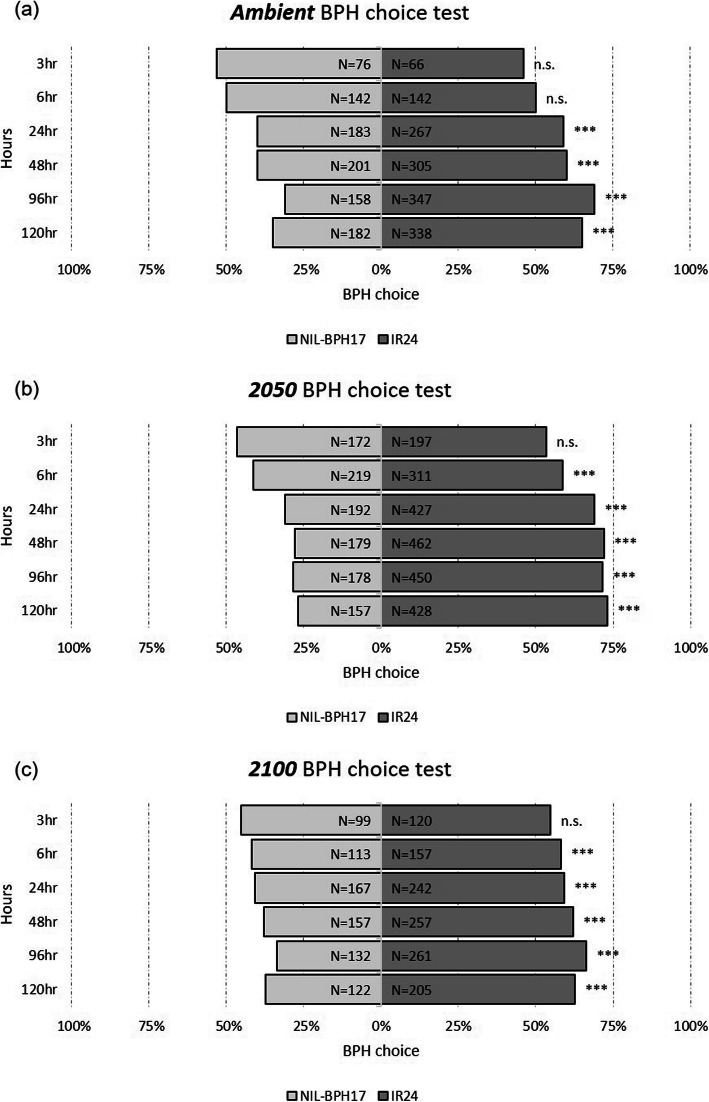
The choice test of *N. lugens* nymphs on IR24 and NIL-*BPH17* under different environments. **a** *Ambient*. **b** *2050*. **c** *2100*. Asterisks indicate differences between IR24 and NIL-*BPH17* as: ***P < 0.001; n.s. means non-significant

**Fig. 8:**
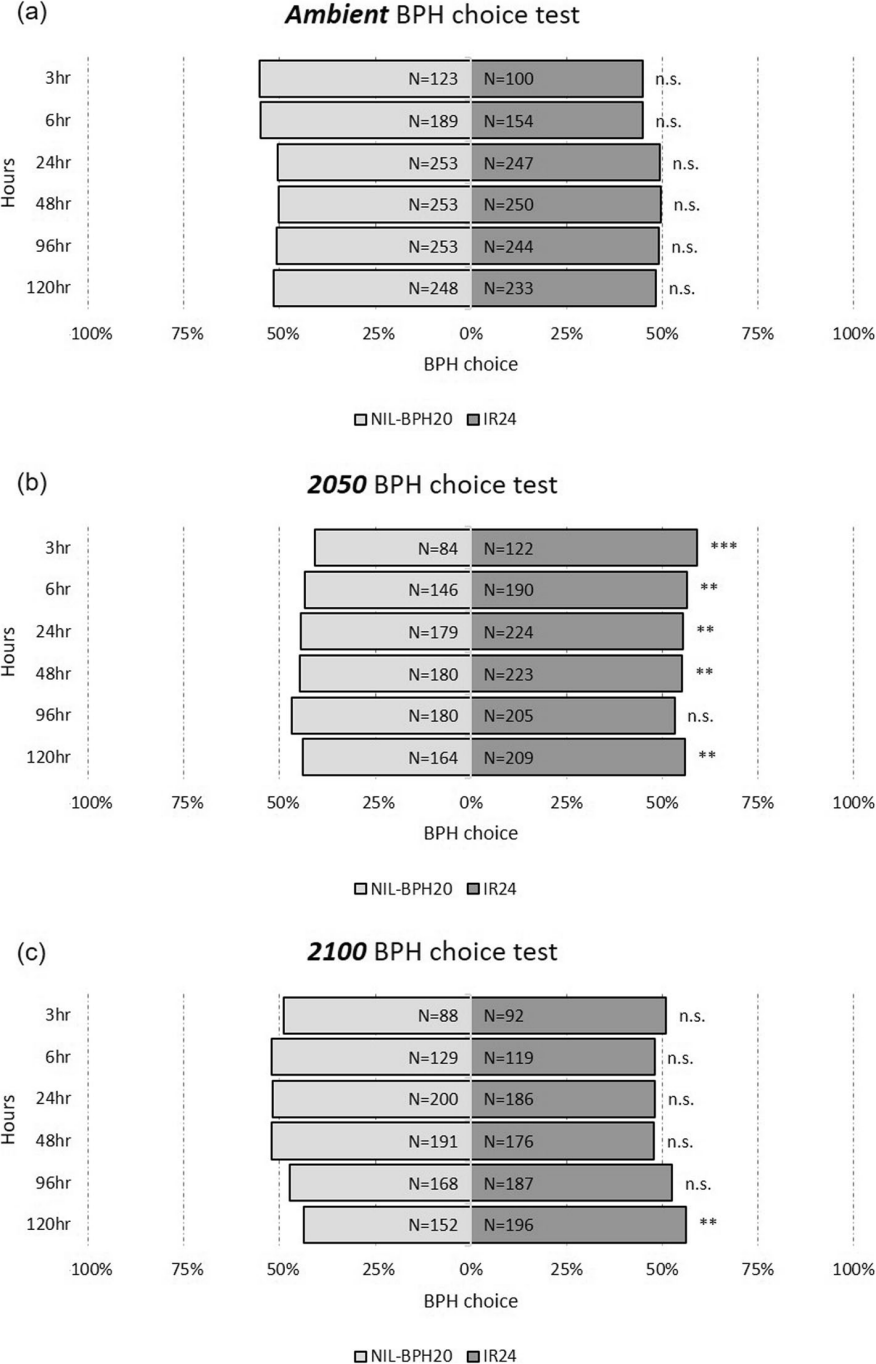
The choice test of *N. lugens* nymphs on IR24 and NIL-*BPH20* under different environments. **a** *Ambient*. **b** *2050*. **c** *2100*. Asterisks indicate differences between IR24 and NIL-*BPH20* as: **P < 0.01; ***P < 0.001; n.s. means non-significant

The oviposition bioassay was examined for *N. lugens* female fecundity and egg hatchability, where *N. lugens* females showed lower fecundity on NIL-*BPH17* than on IR24 and NIL-*BPH20*, except under 2100 (Fig. 6a). Within the NILs, there was no effect on the environmental changes (Fig. 6a), while egg hatchability was affected by environmental changes, where the egg-hatching rate was lower than 2100 than ambient and 2050 (Fig. 6b). There was also no difference among the three varieties (Fig. 6b), indicating that *N. lugens* fecundity is determined by the host plants, which would be unaffected by the environment. However, the environment was the major factor influencing hatchability. In addition to the antibiosis effect, the choice test was tested to understand the antixenosis effect on the NILs. Compared to IR24 and NIL-*BPH17*, more *N. lugens* nymphs chose IR24 instead of NIL-*BPH17,* starting from 24 h to 120 h under the ambient conditions (Fig. 7a). Under 2050 and 2100, *N. lugens* nymphs preferred IR24 at 6 h after the experiment (Fig. 7b and c). However, compared to IR24 and NIL-*BPH20*, there was no difference under the ambient and 2100 conditions (Fig. 8a and c). Under the 2050 conditions, *N. lugens* nymphs preferred IR24 over NIL-*BPH20* at 3 h after the experiment, except at 96 h (Fig. 8b), indicating that NIL-*BPH17* had a strong repellence as the environmental changed, whereas NIL-*BPH20* had a strong repellence only under the 2050 conditions.

## Discussion

The results of this study revealed that the BPH resistance genes would be affected by the environmental changes, in which NILs are valuable genetic resources for identifying potential resistance genes that can resist environmental changes in the decades to come. This study identified NIL-*BPH17* and NIL-*BPH20*, which maintained a low damage score under environmental changes (Table 2) and that *N. lugens* had different responses to those two NILs (Figs. 1, 2, 3, 5, 6a, 7 and 8). The inhibitory effect of NIL-*BPH17* on the *N. lugens* growth and development was unaffected by climate change, whereas NIL-*BPH20* may reduce its resistance during the environmental changes.

The resistance of *BPH17* and *BPH20* was originally screened by the SSST (Rahman et al. 2009; Sun et al. 2005), in which the *BPH17* gene is located on chromosome 4S from the traditional rice germplasm, Rathu Heenati (Sun et al. 2005). *BPH17* was cloned and its function was identified as a cluster of three lectin receptor kinases (*OsLecRK1*-*OsLecRK3*) (Liu et al. 2015). These lectin receptor kinases are localized on the plasma membrane of the vascular bundles of the leaf sheath cells (Liu et al. 2015), where three lectin receptor kinases show distinct expression profiles after *N. lugens* feeding (Liu et al. 2015). In our study, NIL-*BPH17* showed strong resistance (ambient: 2.4; 2050: 2.3; and 2100: 1.3) in the SSST experiment. These results were consistent with another NIL-*BPH17* study under the ambient temperature condition (Nguyen et al. 2019). In addition, the NIL-*BPH17* gene exhibited a strong inhibitory effect on several *N. lugens* parameters, such as in the honeydew assay, PGR, survival rate, fecundity, and choice tests, where our results were consistent with those of (Liu et al. 2015). Surprisingly, in the choice test assay in our study and in the study by (Liu et al. 2015) plants carrying *BPH17* had a lower number of *N. lugens* after 24 h of ambient conditions, indicating that the *BPH17* gene may have strong insect repellent properties. Furthermore, this repellent effect became stronger under the environmental changes, in which the profile of the volatile organic compounds on NIL-*BPH17* should be further examined in terms of their antixenosis effect in the future. Based on our study, the results indicate that *BPH17* may have strong antibiotic and antixenosis effects.

The *BPH20* gene derived from *O. miniuta* is also located on chromosome 4 (Rahman et al. 2009). However, the function of *BPH20* is unclear. In our study, NIL-*BPH20* showed consistent resistance (ambient: 4.0; 2050: 5.7; and 2100: 5.7) in the SSST experiment, but not in the other BPH-relative assays under climate change. Since the SSST assay is a traditional screening method to identify potential BPH-resistant plants, it can mix the antixenosis (non-preference), antibiosis, and tolerance effects. Thus, it would be difficult to characterize the host plant resistance category based on the SSST. *N. lugens* females produced a small amount of phloem-derived excretion by feeding on NIL-*BPH20* under the ambient conditions, where this honeydew bioassay result was consistent with a previous report (Jiang et al. 2018). However, NIL-*BPH20* lost its inhibitory effect under the environmental changes. In addition, *BPH20* did not inhibit several *N. lugens* parameters, such as the PGR, survival rate, fecundity, and hatchability. Moreover, it has been reported that NIL-*BPH20* and the susceptible donor, Taichung 65, showed similar *N. lugens* adult mortality with two *N. lugens* colonies (Nguyen et al. 2019). Thus, these results indicate that NIL-*BPH20* may contain *N. lugens* tolerance and weak antixenosis and antibiotic effects.

Climate change has dramatically changed our society, where it impacts crop production and food and nutrition quality. It is predicted that increasing atmospheric 1 °C would reduce 3.2% crop production in rice (Zhao et al. 2017). Planting insect-resistant varieties is an IPM strategy to reduce the yield loss caused by insect infestation and to reduce pesticide usage. However, in our study, several BPH resistance genes (*BPH4*, *BPH9*, *BPH10*, *BPH18*, *BPH21*, *BPH21*, *BPH32*, *and BPH2 + 32*) would lose their resistance due to climate change. In addition, another three BPH resistant varieties, IR26 (*BPH1*), IR36 (*BPH2*) and IR62 (*BPH26 + 32*), would lose the resistance under elevated temperature (Horgan et al. 2021a; Wang et al. 2010). These results imply that we need to use these insect-resistance traits/genes very carefully to prevent the development of a loss-of-function effect on the insect-resistant varieties in the future. Furthermore, even though NIL-*BPH20* showed a consistent damage score under the environmental changes, *N. lugens* females produced more phloem-derived honeydew on NIL-*BPH20*, implying that *N. lugens* may feed more phloem sap to obtain more nutrients to detoxify the plant’s resistance in the future. In addition, *N. lugens* showed similar growth parameters under the ambient and 2050 conditions (Fig. 6). Thus, the environmental conditions of 2050 will be suitable for *N. lugens*. However, under the environmental conditions of 2100, *N. lugens* would have a lower survival rate and hatchability. This result was consistent with the studies showed that high temperature (34–35 °C, the prediction temperature of 2100) would greatly reduce *N. lugens* survival rate (Horgan et al. 2020; Horgan et al. 2021a; Horgan et al. 2021b; Wang et al. 2010).

Pyramided genes are a strategy to input multiple genes into one plant to synergize the resistance level, which has been used to demonstrate durable insect resistance (Horgan et al. 2019). In our study, two NILs with two pyramided genes (NIL-*BPH18 + 32* and NIL-*BPH9 + 32*) not only showed lower damage scores than NILs with a single BPH gene (*BPH9*, *18*, and *32*) in the ambient environment, but also maintained a consistent resistance under the environmental changes. Thus, the gene pyramiding strategy not only enhances the resistance but also maintains the resistance ability under climate change. In addition, the combination of resistance genes with different protein functions would have a synergistic effect. In our study, it was revealed that each BPH resistance gene (*BPH9*, *18*, and *32*) would have an impact on the environmental changes. *BPH2*, *9*, and *18* are the three of the four alleles of the same locus, which encode a protein with a CC-NB-NB-LRR domain (Zhao et al. 2016), while *BPH32* encodes an unknown short consensus repeat domain (Ren et al. 2016). By using gene pyramiding, a single resistance gene that would have an impact on climate change would maintain/enhance its resistance under the environmental changes. Among the three pyramided gene combinations, the synergistic effect consisted of *BPH18 + 32* = *BPH9 + 32* > *BPH2 + 32*. Thus, it indicates that the alleles at the same locus may have different synergistic effects. Hence, this study provides important contributions to the integration of IPM, where BPH resistance genes may have a strong impact on the environmental changes. By using pyramided genes, the resistance level of the rice plants would be both enhanced and maintained under climate change. Furthermore, the understanding of the impact of the resistance genes under the environmental changes would benefit future host-plant resistance breeding programs that could be conducted on rice against climate change.

## Conclusions

A set of NILs carrying with BPH resistance genes were investigated the responses under climate change impact. Most of tested NILs had changed their resistance level under the environmental changes. In addition, NIL-*BPH17* would maintain its inhibitory effect against *N. lugens* under environmental changes, while NIL-*BPH20* would lose its ability during the environmental changes. These results provide valuable information for future host-plant resistance breeding programs.

## Materials and Methods

### Environmental Chamber Settings

Based on the IPCC prediction (Stocker et al. 2013), three environmental settings in this study were set up as follows: (1) Ambient: 30 °C / 25 °C (light/dark), CO_2_ concentration of 500 ppm; (2) the prediction environment in the year 2050: 32 °C / 27 °C (L/D), CO_2_ concentration of 600 ppm; (3) and the prediction environment in the year 2100 as well as 35 °C /30 °C (L/D) and a CO_2_ concentration of 1000 ppm. The growth chambers were set to a 12:12 h cycle (L:D) under a relative humidity of 55 ± 5%, while the temperature and CO_2_ concentration in the aforementioned settings were further measured as Ambient: 29.67 °C ± 0.02 °C / 24.69 °C ± 0.01 °C (L/D) and a CO_2_ concentration of 531.81 ± 0.58 ppm; 2050: 31.64 °C ± 0.003 °C /26.65 °C ± 0.0001 °C (L/D) and a CO_2_ concentration of 612.19 ± 0.34 ppm; as well as 2100: 34.26 °C ± 0.035 °C /31.95 °C ± 0.015 °C (L/D) and a CO_2_ concentration of 1013.82 ± 30.29 ppm.

### Plant Material

The 12 NILs, of which nine and three NILs carry one and two BPH resistance genes, respectively (Table 1), which were originally obtained from IRRI (Jena et al. 2017). IR24 was obtained from the National Plant Genetic Resources Center at the Taiwan Agricultural Research Institute (TARI), Council of Agriculture (COA), Taiwan. The susceptible check Taichung Native 1 (TN1) for the SSST was obtained from the Taichung District Agricultural Research and Extension Station, COA, Taiwan. Seeds were surface-sterilized with 2% (v/v) NaOCl for 30 min and further washed with distilled water for 10 min. Then, the seeds were germinated on water-moistened paper towels for 2 days under a dark environment at 30 °C.

### Insect Rearing

The *N. lugens* biotype 1 colony was originally obtained from the Chiayi Agricultural Experiment Station, TARI, COA, Taiwan, in which the *N. lugens* colonies were mass-reared on the TN1 seedlings in fine-meshed insect cages (BugDorm-4, Megaview, Taichung, Taiwan). The TN1 seedlings were placed in trays and treated with soluble fertilizer consisting of 120, 40, and 60 kg/ha of nitrogen (N), phosphorus (P) and potassium (K) until the 4-leaf stage, which was periodically changed to maintain the *N. lugens* colonies. For this study, the *N. lugens* colonies were reared separately in three different environmental chambers reflecting the ambient, 2050, and 2100 environments, which were maintained for at least two generations prior to the experiment. Each *N. lugens* colony was only used for the corresponding environmental setting. After the experiment, the insects were not used to maintain the colony or used in other experiments.

### The Standard Seed-Box Screening Test (SSST)

In this study, the SSST was used to evaluate the plant’s resistance against *N. lugens*. Briefly, 20 seeds of each NIL, IR24, and susceptible check TN1 were sown in rows. Then, 14 days after sowing, the seedlings were infested with 2nd to 3rd instar *N. lugens* nymphs at a density of 10 nymphs per seedling. The damage levels of the tested plants were evaluated based on the standard evaluation system (IRRI 2013) when the susceptible TN1 plants were declared dead. This experiment was repeated three times in each environmental setting.

### Honeydew Excretion Test

The amount of honeydew in the *N. lugens* feeding on the tested plants was used for the antibiosis resistance test. Because of the unique *N. lugens*-feeding behavior, the honeydew excretion can be determined by the method of using filter paper that was treated with bromocresol green (Pathak and Heinrichs 1982). The NIL-*BPH17*, NIL-*BPH20*, and the background variety IR24 were chosen for the honeydew excretion test. Briefly, seedlings were transferred into plastic pots with a 64 mm base diameter, a 95 mm aperture diameter, and a height of 165 mm that contained paddy soil and was treated with a soluble fertilizer. After 30 days of germination, the branches that emerged from the main tiller of the tested plants were removed and a plastic layer with a hole in the middle was placed on top of the plants’ soil surface. The filter paper that was treated with 0.1% bromocresol green (Alfa Aesar, Haverhill, MA, USA) was placed on top of the plastic layer, covered with a plastic cover that had a hole in the middle, and secured with a cotton plug. One female gravid *N. lugens* with 2 h starvation treatment was placed on the plant to allow its feeding for 24 h. Next, the filter papers were collected and scanned. Due to the different chemical properties of the phloem and xylem, the bromocresol green indicates the phloem-derived excretion as blue-rimmed spots and the xylem-derived excretion as transparent-rimmed spots (Auclair et al. 1982; Kimmins 1989). The area of each spot was measured using the ImageJ software (Rasband 1997). The sample size (n) of the replicates (N) in each environmental setting reflecting the NIL-*BPH17* and IR24 experiment as well as the NIL-*BPH20* and IR24 experiment, which both consisted of the ambient, 2050, and 2100 environmental settings of: *N* = 3 and *n* = 8–12, respectively.

### *N. lugens* Population Growth Rate (PGR)

The seedlings were transferred into plastic pots that contained paddy soil and were treated with a soluble fertilizer. At 35 days after germination, the branches, with the exception of the main tiller of the tested plants, were removed. Then, 10 weighted 2nd instar *N. lugens* nymphs were placed on the plant with a plastic cover that had a hole in the middle and was secured with a cotton plug. After the 4-day infestation, the surviving *N. lugens* nymphs were counted and weighed, in which the formula for the PGR was as follows (Edwards 2001; Klingler et al. 2005):

$$ \mathrm{PGR}=\frac{\log \left(\mathrm{Survived}\ N. lugens\ \mathrm{nymph}\ \mathrm{weight}/\mathrm{Survived}\ N. lugens\ \mathrm{nymph}\ \mathrm{number}\right)-\log \left(\mathrm{Total}\ N. lugens\ \mathrm{nymph}\ \mathrm{weight}/\mathrm{total}\ N. lugens\ \mathrm{nymph}\ \mathrm{number}\right)}{\mathrm{Days}} $$

The sample size (n) of the replicates (N) in each environmental setting in the NIL-*BPH17*, NIL-*BPH20*, and IR24 experiment included *N* = 3 and *n* = 4–5 across all three environmental settings.

### *N. Lugens* Survival Rate

The seedlings were transferred into plastic pots that contained paddy soil and were treated with the soluble fertilizer. At 30 days after germination, the branches, with the exception of the main tiller of the tested plants were removed. Then, ten 3rd instar *N. lugens* nymphs were placed on the plant with a plastic cover that had a hole in the middle and was secured with a cotton plug. The survival rate of *N. lugens* nymphs was recorded until 9 days after infestation. The sample size (n) of the replicates (N) in each environmental setting for the NIL-*BPH17*, NIL-*BPH20*, and IR24 experiment included N = 3 and *n* = 2–5 across all three environmental settings.

### *N. Lugens* Choice Test

The choice test was used as the non-preference test, where five plants per IR24, NIL-*BPH17*, IR24, and NIL-*BPH20* line were cross-planted in pots of a 160 mm base diameter, a 200 mm aperture diameter, and a height of 222 mm that contained paddy soil and were treated with a soluble fertilizer. A total of 100 3rd instar nymphs were placed on a Petri dish that was transferred to the center of the pot when the plants were at the 4-leaf stage, which were covered with fine-meshed insect cages (BugDorm-4, Megaview, Taichung, Taiwan). After opening the top of the Petri dish, the number of nymphs on each plant was counted at 3, 6, 24, 48, 96, and 120 h, where the total number of nymphs on NIL-*BPH17* & IR24 and NIL-*BPH20* & IR24 were calculated. The sample size (n) in each environmental setting for the NIL-*BPH17* and IR24 experiment consisted of the ambient: *n* = 600; 2050: *n* = 900; and 2100: *n* = 500, while in the case of the NIL-*BPH20* and IR24 experiment, the values included the ambient: n = 600; 2050: n = 600; and 2100: *n* = 500.

### *N. Lugens* Fecundity and the Hatchability of Eggs

The seedlings were transferred into plastic pots that contain paddy soil and were treated with a soluble fertilizer. At 30 days after germination, the branches, with the exception of the main tiller of the tested plants, were removed. One female gravid *N. lugens* and one male *N. lugens* were transferred to the plant with a plastic cover that had a hole in the middle and was secured with a cotton plug. Adult insects were removed after the fifth day of the experiment, while the newly hatched nymphs were further counted for the following 10 days. At the end of the experiment at 45 days after germination, the leaf sheath was cut and counted to obtain the number of non-hatched eggs. The sample size (n) of the replicates (N) in each environmental setting for the NIL-*BPH17*, NIL-*BPH20*, and IR24 experiment included *N* = 3 and *n* = 2–6 across all three environmental settings.

### Statistical Analysis

For the SSST, the data were analyzed using a two-way analysis of variance (ANOVA). the honeydew test, PGR, fecundity, and the hatchability of eggs were analyzed using a one-way ANOVA. The least significant difference test was used to test for differences at *P* < 0.05, while the choice test was analyzed using the *z*-test. All data were analyzed using R software (v3.5.0) (R Core Team 2013).

## Data Availability

The datasets used and/or analyzed during the current study are available from the corresponding author on reasonable request.

## Abbreviations

BPH: Brown planthopper
CO_2_: Carbon dioxide
IPCC: The Intergovernmental Panel on Climate Change
IPM: Integrated pest management
IRRI: International Rice Research Institute
NIL: Near-isogenic line
SST: The standard seed-box screening test
